# Supplementary material for: A diverse intrinsic antibiotic resistome from a cave bacterium
Source: Nat Commun. 2016 Dec 8;7:13803. doi: 10.1038/ncomms13803 (PMC5155152; doi:10.1038/ncomms13803)
Supplement: Supplementary Information — Supplementary Figures and Supplementary Tables. [file ncomms13803-s1.pdf]

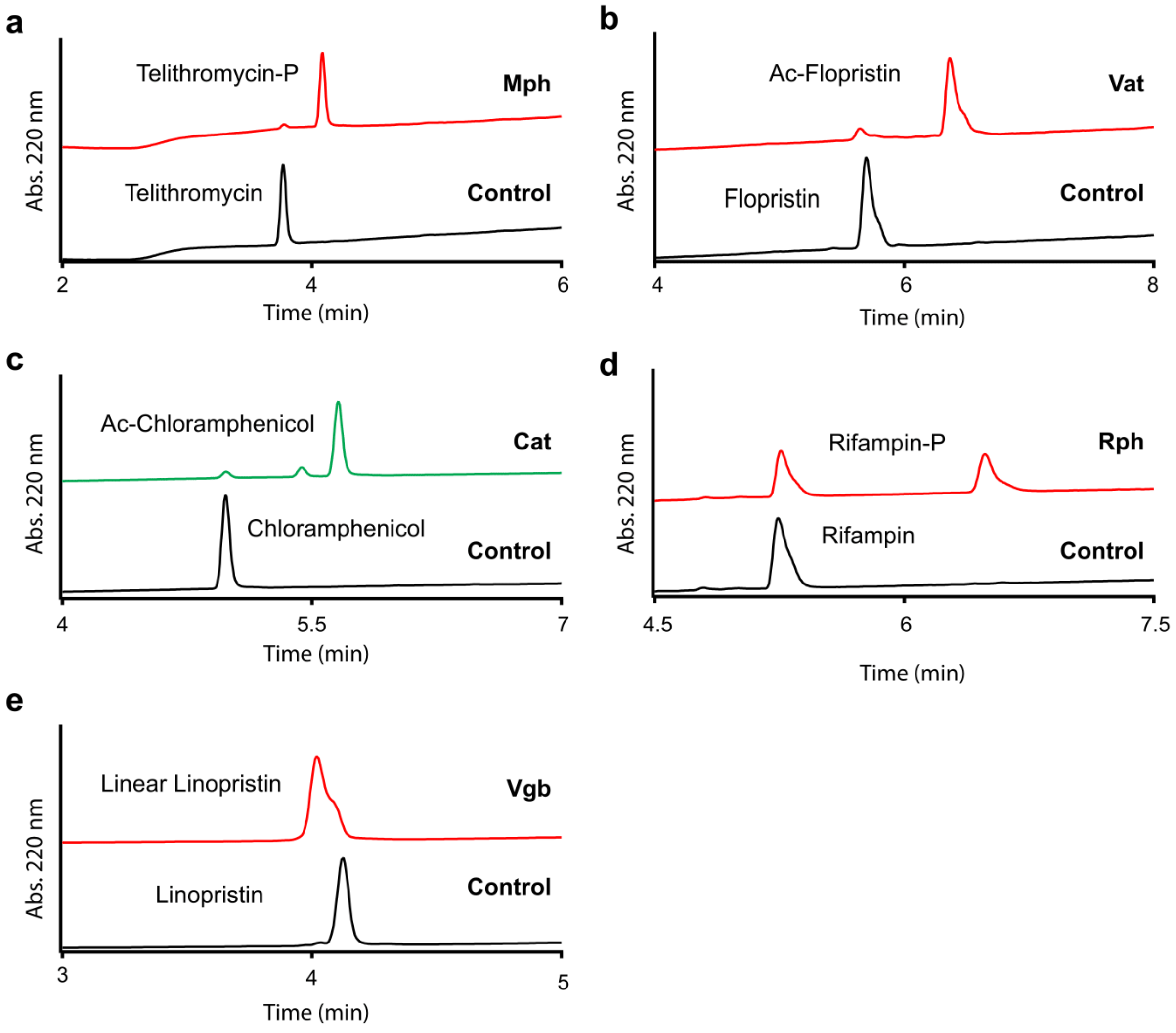

Supplementary Figure 1: Functional validation of antibiotic-inactivating enzymes identified in this study. Enzymatic reactions were analyzed using HPLC and confirmed to modify their predicted substrates.

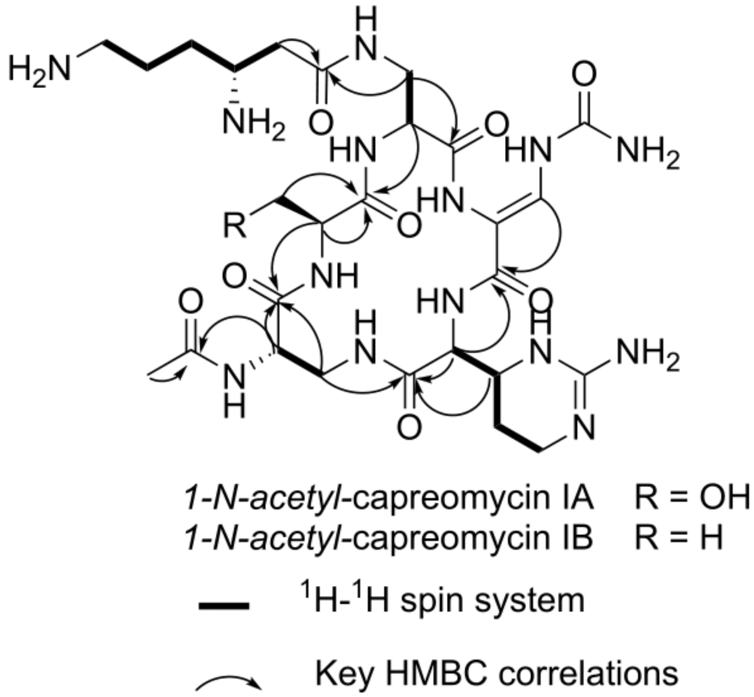

Supplementary Figure 2: Key TOCSY, COSY and HMBC correlations of *1-N-acetyl*-capreomycin IA and *1-N-acetyl*-capreomycin IB

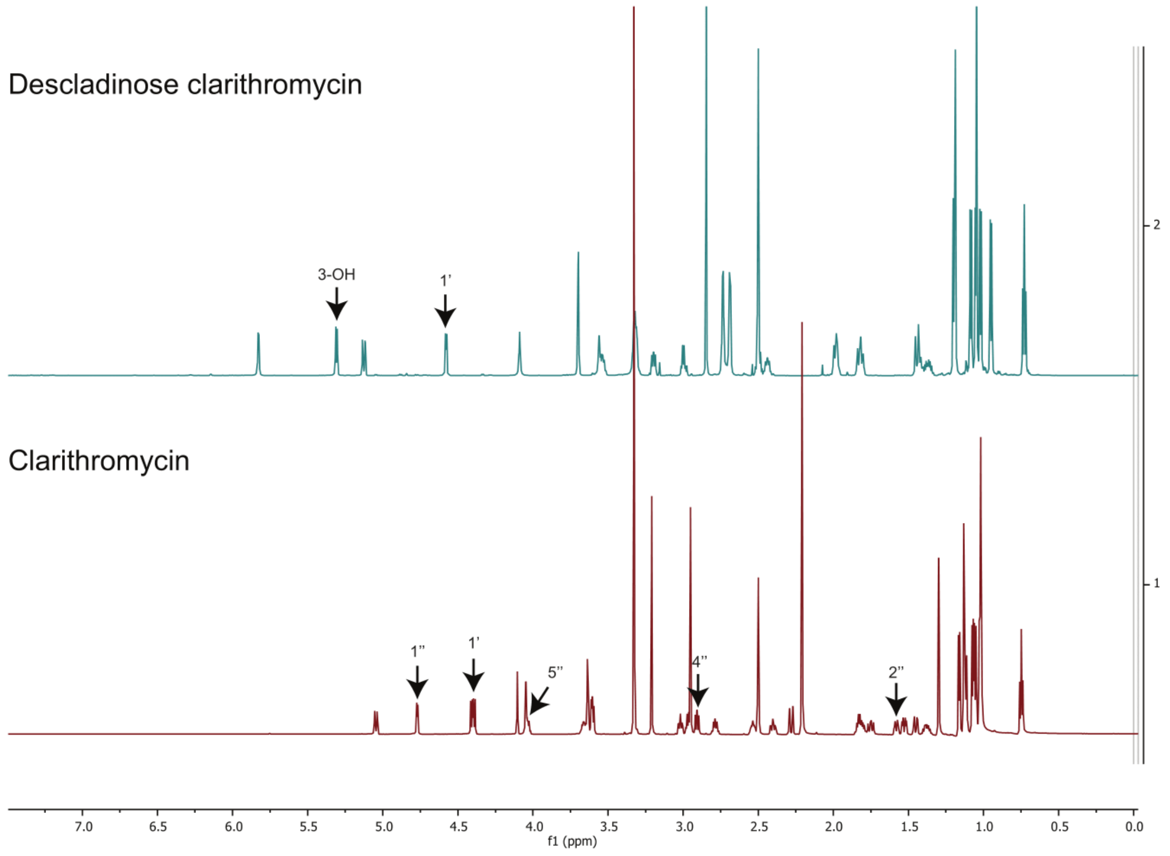

Supplementary Figure 3: Comparison of 1H NMR spectra of clarithromycin and descladinose clarithromycin in DMSO-d6.

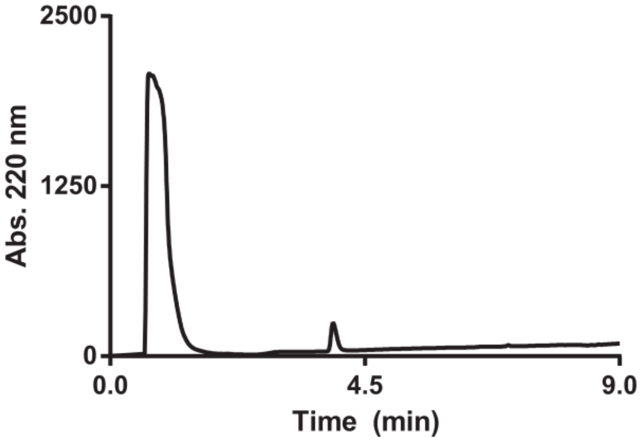

Supplementary Figure 4: Descladinose clarithromycin analyzed by HPLC.

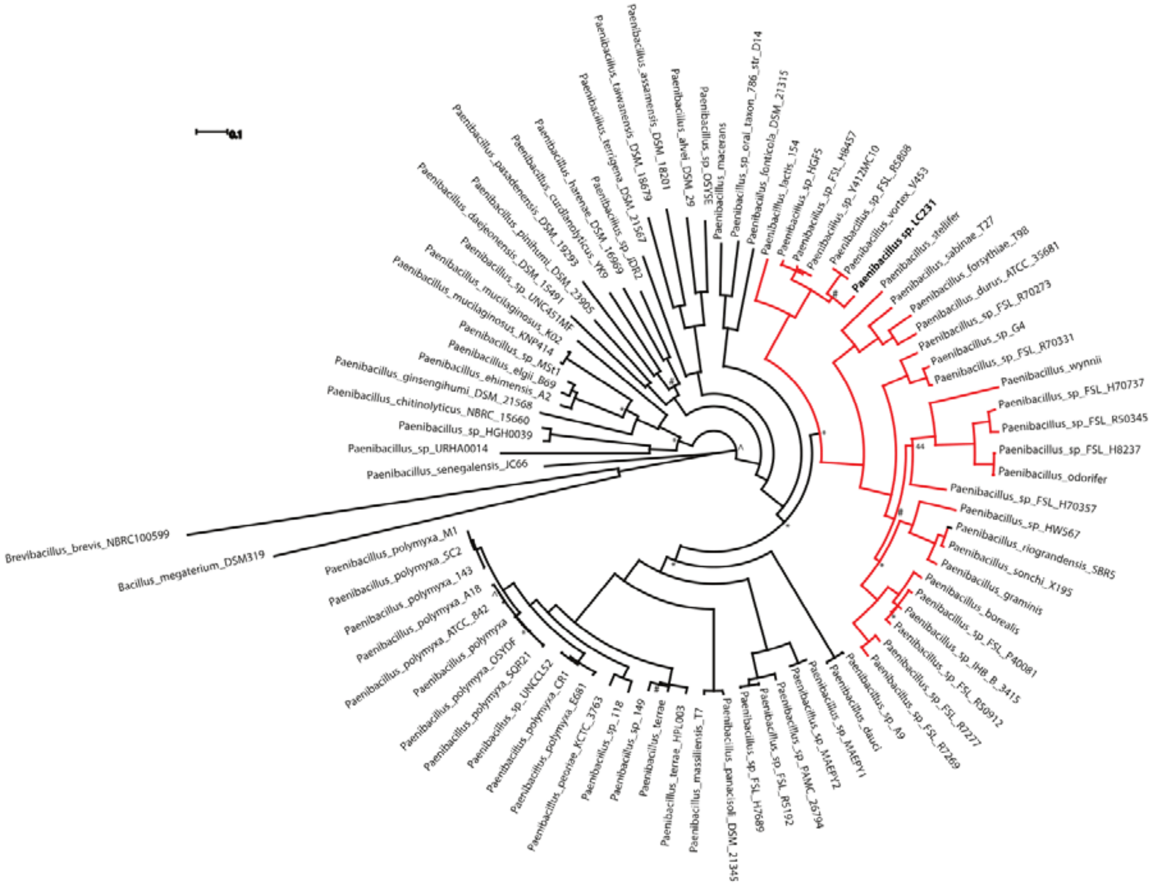

Supplementary Figure 5: *Paenibacillus* species tree presented as a circular cladogram. Concatenated maximum-likelihood tree using 10 housekeeping genes (*dnaA*, *gyrB*, *pyrH*, *recA*, *pgi*, *trpB*, *atpD*, *sucC*, *rpoB*, *topA*) derived from *Paenibacillus* genome sequences. The segment highlighted in red was used for the pan-*Paenibacillus* comparison of resistance enzymes. Unless otherwise noted, bootstrap values are 100. The following symbols represent bootstrap values; >80 (*), >70 (#) and >50 (^).

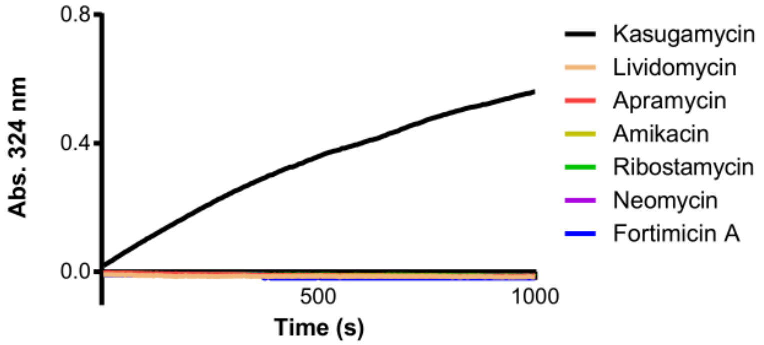

Supplementary Figure 6: Substrate specificity of AAC(2')-IIb. AAC(2')-IIb specifically acetylates kasugamycin in an *in vitro* acetyltransferase assay and does not modify other aminoglycosides.

Supplementary Table 1: Quantitative antibiogram of *Paenibacillus* sp. LC231, results of antibiotic inactivation assays and resistance gene prediction using the Resistance Gene Identifier (RGI).

Antibiotic	MIC (µg/mL)	Antibiotic Inactivation?	RGI Prediction	Functional genomics
Meropenem	2	No		
Cefotaxime	2	n/a		
Cephalexin	≥128	No		
Ampicillin	8	No		
Teicoplanin	0.25	n/a		
Vancomycin	1	No		
Bacitracin	≥128	**Yes**		*bahA*
Fosfomycin	≥128	No		
Polymyxin B	8	n/a		
Gramicidin D	1	n/a		
Daptomycin	16	**Yes**		
Rifampin	1	**Yes**	*rph, rpoB* A473T	
Novobiocin	0.25	n/a		
Mupirocin	≥100	No		*ileRS*
Tylosin	16	**Yes**		
Spiramycin	≥128	**Yes**		
Telithromycin	2	**Yes**	*mph*	
Erythromycin	2	No		
Azithromycin	16	No		
Clarithromycin	0.25	No		
Linopristin	64	**Yes**	*vgb*	
Clindamycin	16	No	*clPa (cfr-like)*	*llmA*
Flopristin	32	**Yes**	*clPa (cfr-like), vat*	
Tiamulin	32	No	*clPa (cfr-like)*	*taeA*
Neomycin	2	n/a	*ant(4')-I*	*aac(6')*
Gentamicin	2	n/a	*ant(4')-I*	*aac(6')*
Apramycin	8	n/a		
Amikacin	16	n/a		*aac(6')*
Tobramycin	32	No	*ant(4')-I*	*aac(6')*
Spectinomycin	≥128	n/a		
Kasugamycin	≥128	No		*aac(2')-IIa*
Kanamycin	≥128	No	*ant(4')-I*	*aac(6')*
Streptomycin	≥128	No	*aadK, aph(3'')-I*	
Linezolid	1	n/a	*cfr*	
Tetracycline	≥128	No		*tetAB(48)*
Capreomycin	32	No		*cpaA*
Thiostrepton	0.25	No		
Chloramphenicol	32	**Yes**	*cat*	
Fusidic acid	8	No		
Factumycin	16	n/a		
Nalidixic acid	4	n/a		
Ciprofloxacin	0.25	n/a		
Levofloxacin	0.25	n/a		

Supplementary Table 2: Quantitative antibiograms of *Staphylococcus aureus* RN4220, *Kocuria rhizophila*, and *Paenibacillus lautus* ATCC 43898**.** Value units are µg/mL.

**Antibiotic**	***Paenibacillus lautus* ATCC 43898** MIC (µg/mL)	***S. aureus* RN4220** MIC (µg/mL)	***K. rhizophila*** MIC (µg/mL)
Meropenem	2	0.06	0.06
Cefotaxime	2	1	0.03
Cephalexin	64	2	0.03
Ampicillin	4	0.5	0.02
Teicoplanin	0.25	1	2
Vancomycin	0.5	1	0.5
Bacitracin	≥128	16	1
Fosfomycin	≥128	1	2
Polymyxin B	2	32	32
Gramicidin D	32	8	1
Daptomycin	4	2	0.25
Rifampin	2	0.02	0.02
Novobiocin	0.25	0.25	0.25
Mupirocin	≥100	0.2	100
Tylosin	8	1	0.125
Spiramycin	32	2	0.25
Telithromycin	0.5	0.06	0.02
Erythromycin	0.5	0.25	0.03
Azithromycin	4	0.5	0.06
Clarithromycin	0.125	0.125	0.02
Fusidic acid	2	0.125	0.5
Linopristin	32	4	4
Clindamycin	8	0.125	0.125
Flopristin	64	0.02	0.02
Neomycin	0.5	1	0.5
Gentamicin	2	1	1
Apramycin	16	2	16
Amikacin	16	4	2
Tobramycin	16	0.5	4
Spectinomycin	≥128	32	32
Kasugamycin	≥128	128	128
Kanamycin	64	2	4
Streptomycin	≥128	8	32
Linezolid	1	0.5	0.5
Tetracycline	8	0.25	1
Capreomycin	32	256	256
Thiostrepton	0.5	0.25	0.25
Chloramphenicol	32	2	0.5
Factumycin	16	32	1.25
Tiamulin	128	0.5	0.125
Ciprofloxacin	0.25	-	-
Levofloxacin	0.25	-	-
Nalidixic acid	4	-	-

Supplementary Table 3: Comparison of antibiotic resistance determinants with their closest ortholog and results from RGI prediction of the *Paenibacillus* sp. LC231 genome.

Resistance Enzyme	Closest relative [a] (% ID)	Host	Location	RGI results (e-value)[b]
AAC(2')-IIb	AAC(2')-IIa (31%)	*Acidovorax avenae*	Mobile element	No hit
AAC(6')-34	AAC(6')-Im (39.8%)	*Enterococcus faecium*	Mobile element	No hit
CpaA	None			-
VatI	VatG (64.3%)	*Enterococcus faecium*	Mobile element	8.19×10^{-100}
VgbC	VgbA (58.6%)	*Staphylococcus aureus*	Mobile element	5.27×10^{-126}
CatU	CatQ (53.4%)	*Clostridium perfringens*	Chromosome	3.19×10^{-74}
MphI	MphB (42.6%)	*E. coli*	Mobile element	4.12×10^{-94}
RphB	RphWAC4747 (56.3%)	*Streptomyces sp. WAC4747*	Chromosome	0
IleRS	MupB (49.9%)	*Staphylococcus aureus*	Mobile element	No hit
BahA	None			-
LlmA	None			-
TetAB(48)	DrrA (55.2%) DrrB (43.0%)[c]	*Streptomyces peucetius*	Chromosome	No hit
TaeA	None			-

a- Percent identity is from pair-wise comparison of protein sequences.
b- An e-value of $< 1.0 \times 10^{-50}$ was required in order to be considered a hit.
c- The substrate of DrrAB is daunorubicin and doxorubicin and is not known to export tetracyclines.

Supplementary Table 4: Mass characterization of antibiotics modified by resistance enzymes

Resistance enzyme	Antibiotic	Mechanism	Mode	*m/z* (substrate)	*m/z* (product)	Mass difference
AAC(2')-IIb	Kasugamycin	Acetylation	Positive	380.3	422.2	41.9
AAC(6')-34	Sisomicin	Acetylation	Positive	448.4	490.3	41.9
CpaA	Capreomycin IA	Acetylation	Positive	669.4	711.4	42.0
VatI	Flopristin	Acetylation	Positive	532.4	574.3	41.9
VgbC	Linopristin	β-elimination	Negative	948.6	948.6	0.0
CatU	Chloramphenicol	Acetylation	Negative	320.9	363.0	42.1
MphI	Telithromycin	Phosphorylation	Positive	812.6	892.7	80.1
RphB	Rifampin	Phosphorylation	Positive	823.1	903.1	80.0
BahA	Bacitracin	Amidohydrolysis	Positive	1422.7572	1423.7416	0.9844

Supplementary Table 5: Tandem mass spectrometry analysis of bacitracin and inactivated bacitracin.

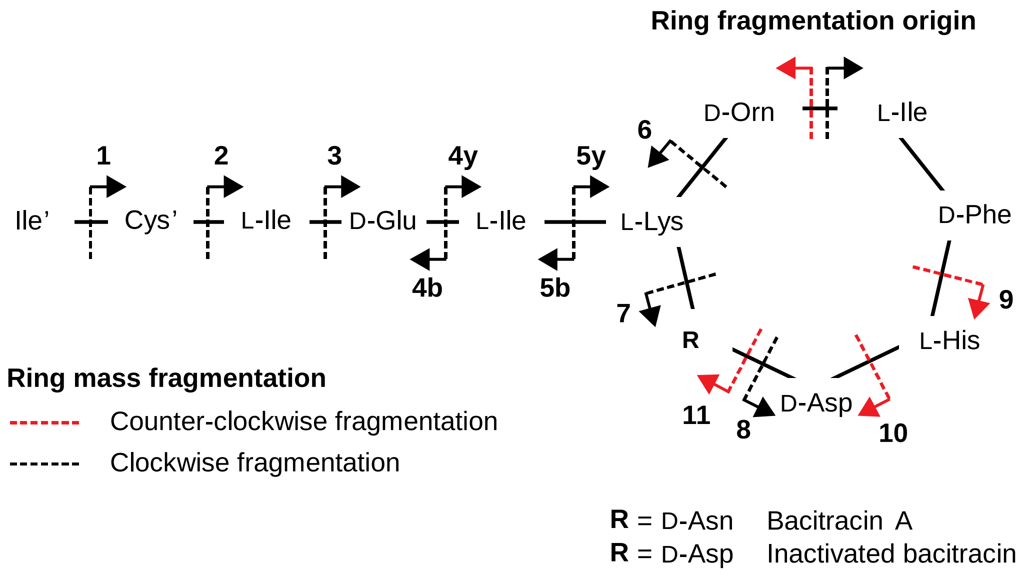

Ion	Bacitracin	Inactivated bacitracin	δ mass
Intact	1422.7642	1423.7486	0.9844
**MS^2**			
1	1337.53	1338.52	0.99
2	1224.62	1225.56	0.94
3	1111.53	1112.50	0.97
4y	982.55	983.50	0.95
4b	441.19	441.20	0.01
5y (ring)	869.46	870.44	0.98
5b	554.39	554.40	0.01
**MS^3 (ring)**			
6	754.47	755.46	0.99
7	627.28	628.26	0.98
8	513.41	513.40	-0.01
9	609.38	610.38	1.00
10	472.41	473.37	0.96
11	357.38	358.42	1.04

Supplementary Table 6: Steady-state kinetics of antibiotic-modifying enzymes.

**Enzyme**	**Substrate**	**K_m (μM)**	**k_{cat} (s^{-1})**	**K_i (μM)**	**k_{cat}/K_m($s^{-1}M^{-1}$)**
CatU					
	Chloramphenicol	50 ± 3	-		-
	Acetyl-CoA	400 ± 20	94 ± 2		2.3×10^5
VgbC					
	Linopristin	4.8 ± 1.4	1.2 ± 0.08		2.5×10^5
MphI					
	Tylosin	2.9 ± 0.9	0.29 ± 0.01		1.0×10^5
	Spiramycin	24.4 ± 6.8	1.2 ± 0.1	475 ± 161	4.9×10^4
	Telithromycin	8.3 ± 1.6	0.45 ± 0.02		5.2×10^4
	GTP	10.0 ± 1.8	0.40 ± 0.01		4.0×10^4
CpaA					
	Capreomycin	0.76 ± 0.12	0.51 ± 0.04	23 ± 7	6.8×10^5
	Acetyl-CoA	39 ± 3	0.49 ± 0.01		1.3×10^4
VatI					
	Linopristin	4.8 ± 0.5	3.10 ± 0.10		6.4×10^5
	Acetyl-CoA	48 ± 3	4.1 ± 0.1		8.6×10^4
RphB					
	Rifampin	0.44 ± 0.13	0.19 ± 0.01		4.3×10^5
	ATP	54 ± 10	0.17 ± 0.01		3.1×10^3
AAC(2’)-IIb					
	Kasugamycin	44 ± 2	2.95 ± 0.03		6.7×10^4
	Acetyl-CoA	13 ± 1	2.9 ± 0.1		3.1×10^3
AAC(6’)-34					
	Sisomicin	4.7 ± 0.4	0.65 ± 0.06	213 ± 25	1.4×10^5
	Acetyl-CoA	220 ± 26	0.39 ± 0.02		1.8×10^3

Supplementary Table 7: ^{1}H and ^{13}C NMR Data of capreomycin IA and *1-N-acetyl*-capreomycin IA in D_2O.

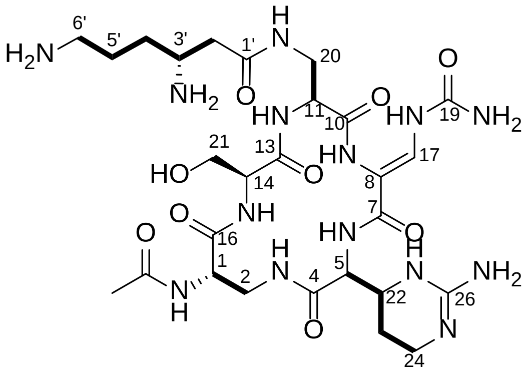

	Capreomycin IA		*1-N-acetyl*-Capreomycin IA		
**No.**	***δ*H**	***δ*C**	***δ*H**	***δ*C**	**Δppm(*δ*H)**
1	4.37 (dd, *J* = 10.1, 5.3 Hz)	50.9	4.60 (dd, *J* = 10.5, 4.9 Hz)	52.0	0.23
2	3.24 (dd, *J* = 13.6, 10.1 Hz)<br>4.09 (dd, *J* = 13.6, 5.3 Hz)	39.4	3.02-3.06 (m, overlapped)<br>3.82-3.88 (m, overlapped)	40.3	-0.20<br>-0.24
4		171.1		171.0	
5	5.01 (d, *J* = 2.1 Hz)	55.1	5.00 (d, *J* = 2.1 Hz)	54.8	-0.01
7		167.4		167.3	
8		104.9		104.7	
10		172.1		171.7	
11	4.41-4.54 (m, overlapped)	53.3	4.26-4.35 (m, overlapped)	53.4	-0.17
13		172.2		172.6	
14	4.81-4.84 (m, overlapped)	54.6	4.72-4.78 (m, overlapped)	54.2	-0.07
16		167.4		171.3	
17	8.03 (s)	135.2	8.02 (s)	135.2	-0.01
19		156.4		156.6	
20	3.73-3.78 (2H, m, overlapped)	38.0	3.70-3.82 (2H, m, overlapped)	37.9	-0.03
21	3.82-3.88 (2H, m, overlapped)	62.3	3.69-3.89 (2H, m, overlapped)	62.4	-0.01
22	4.47 (m)	49.0	4.47 (m)	49.1	0
23	2.05-2.12 (m, overlapped)<br>1.67-1.75 (m, overlapped)	22.7	2.04-2.10 (m, overlapped)<br>1.63-1.71 (m, overlapped)	22.9	-0.02<br>-0.04
24	3.29-3.33 (m, overlapped)	36.1	3.30-3.33 (m, overlapped)	36.1	-0.01
26		154.4		154.4	
1’		172.2		172.1	
2’	2.69 (dd, *J* = 16.2, 8.3 Hz)<br>2.80 (dd, *J* = 16.2, 7.3 Hz)	36.6	2.61-2.68 (m, overlapped)<br>2.69-2.79 (m, overlapped)	36.7	-0.04<br>-0.04
3’	3.65-3.71 (m, overlapped)	48.3	3.61-3.69 (m, overlapped)	48.2	-0.03
4’	1.75-1.87 (2H, m, overlapped)	29.0	1.73-1.82 (2H, m, overlapped)	29.1	-0.03
5’	1.75-1.87 (2H, m, overlapped)	22.7	1.73-1.82 (2H, m, overlapped)	22.9	-0.03
6’	3.04-3.08 (2H, m, overlapped)	38.8	3.01-3.05 (2H, m, overlapped)	38.8	-0.03
C<u>H</u>$_3$C=O			2.03 (s)	21.5	
CH$_3$<u>C</u>=O				174.0	

Supplementary Table 8: ^{1}H and ^{13}C NMR Data of capreomycin IB and *1-N-acetyl*-capreomycin IB in D_2O.

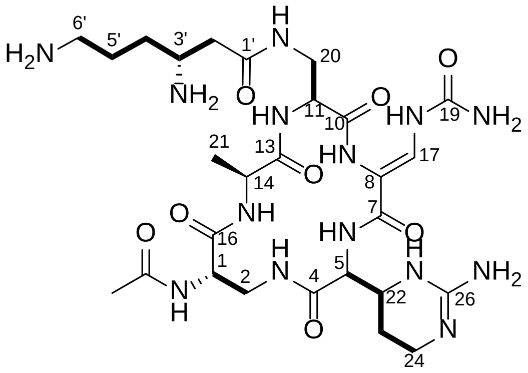

	Capreomycin IB		*1-N-acetyl*-Capreomycin IB		
**No.**	***δ*H**	***δ*C**	***δ*H**	***δ*C**	**Δppm(*δ*H)**
1	4.33 (dd, *J* = 7.9, 5.1 Hz)	51.1	4.56 (dd, *J* = 9.1, 4.9 Hz)	51.9	0.23
2	3.31 (m, overlapped)<br>4.12 (dd, *J* = 14.1, 5.1 Hz)	39.4	3.05-3.09 (m, overlapped)<br>3.82-3.88 (m, overlapped)	40.3	-0.24<br>-0.27
4		171.1		171.0	
5	4.98 (d, *J* = 2.1 Hz)	55.0	4.97 (d, *J* = 2.1 Hz)	54.8	-0.01
7		167.1		167.2	
8		104.9		104.7	
10		172.0		171.9	
11	4.41-4.54 (m, overlapped)	53.1	4.26-4.35 (m, overlapped)	53.1	-0.17
13		175.6		175.7	
14	4.65 (t, *J* = 7.3 Hz)	48.7	4.64 (t, *J* = 7.3 Hz)	48.1	-0.01
16		166.7		171.3	
17	8.03 (s)	135.0	8.02 (s)	135.1	-0.01
19		156.4		156.6	
20	3.67-3.71 (m, overlapped)<br>3.82-3.86 (m, overlapped)	37.9	3.64-3.69 (m, overlapped)<br>3.84-3.88 (m, overlapped)	37.8	-0.02<br>-0.02
21	1.42 (3H, d, *J* = 7.3 Hz)	18.3	1.37 (3H, d, *J* = 7.3 Hz)	18.3	-0.05
22	4.39 (m)	49.0	4.41 (m)	48.9	-0.02
23	2.05-2.12 (m, overlapped)<br>1.67-1.75 (m, overlapped)	22.8	2.04-2.10 (m, overlapped)<br>1.63-1.71 (m, overlapped)	22.8	-0.02<br>-0.04
24	3.29-3.33 (m, overlapped)	36.1	3.30-3.33 (m, overlapped)	36.1	-0.01
26		154.4		154.4	
1’		172.0		172.1	
2’	2.69 (dd, *J* = 16.2, 8.3 Hz)<br>2.77 (dd, *J* = 16.2, 5.1 Hz)	36.6	2.61-2.68 (m, overlapped)<br>2.69-2.79 (m, overlapped)	36.7	-0.04<br>-0.04
3’	3.65-3.71 (m, overlapped)	48.3	3.61-3.69 (m, overlapped)	48.2	-0.03
4’	1.75-1.87 (2H, m, overlapped)	29.0	1.73-1.82 (2H, m, overlapped)	29.1	-0.03
5’	1.75-1.87 (2H, m, overlapped)	22.7	1.73-1.82 (2H, m, overlapped)	22.9	-0.03
6’	3.04-3.08 (2H, m, overlapped)	38.8	3.01-3.05 (2H, m, overlapped)	38.8	-0.03
C<u>H</u>$_3$C=O			2.02 (s)	21.5	
CH$_3$<u>C</u>=O				174.0	

Supplementary Table 9: ^{1}H and ^{13}C NMR spectra of clarithromycin and descladinose clarithromycin in DMSO-d6.

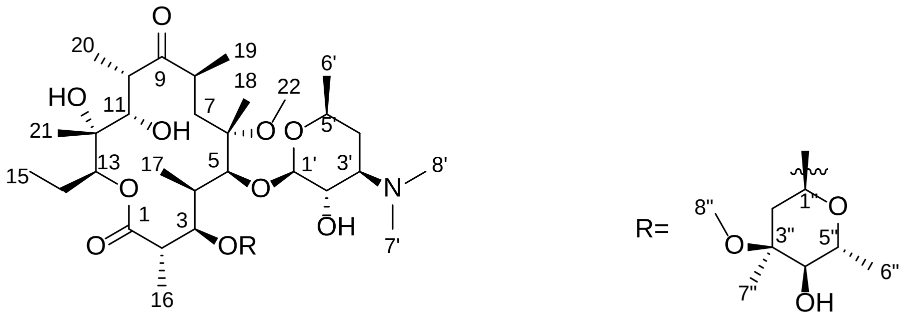

	Clarithromycin (R=cladinose)		Descladinose clarithromycin (R=H)	
**No.**	***δ*H**	***δ*C**	***δ*H**	***δ*C**
1		175.2	-	174.8
2	2.8 (dt, J = 9.4, 7.2 Hz)	44.5	2.48 (m)	44.5
3	3.63 (m)	79.4	3.21 (m)	75.6
3-OH	-	-	5.32 (d, J = 6.7 Hz)	-
4	1.84 (m)	38.7	1.99 (m)	36.1
5	3.62 (m)	79.4	3.7 (d, J = 2.5 Hz)	80.6
6	-	72.6	-	77.5
7	1.76 (m)<br>1.46 (m)	38.5	1.8 (m)<br>1.42 (m)	37.9
8	2.56 (m)	39.9	2.47 (m)	39.9
10	2.54 (m)	43.7	2.43	43.7
11	3.63 (m)	68.9	3.53 (m)	67.4
12	-	74.2	-	74.2
13	5.05 (dd, J= 11.2, 2.3 Hz)	76.1	5.13 (dd, J = 11.2, 2.5 Hz)	75.6
14	1.83 (m)<br>1.36 (m)	20.8	1.8 (m)<br>1.34 (m)	21
15	0.72 (t, J = 7.4 Hz)	10.4	0.77 (t, J = 7.4 Hz)	10.4
16	1.13 (d, J = 7.4 Hz)	15.8	1.1	15.6
17	1.01 (d, J = 6.5 Hz)	12.3	1.02 (d, J = 6.5 Hz)	12.3
18	1.05 (s)	18.6	1.05 (d, J = 6.3 Hz)	17.7
19	1.11 (d, J = 7.4 Hz)	17.8	1.06 (d, J = 6.3 Hz)	15.5
20	0.95 (d, J = 7.4 Hz)	8.4	0.95 (d, J = 7.4 Hz)	8.4
21	1.2 (s)	19.3	1.19 (s)	20.7
22	3.26 (s)	49.03	2.85 (s)	49.8
1’	4.37 (dd, J = 12.7, 7.4 Hz)	102.3	4.57 (d, J = 6.7 Hz)	100.4
2’	3.02 (ddd, J = 10.1, 7.3, 2.5 Hz)	70.5	3.33 (m)	69.4
2’-OH	4.1(s)		4.07 (s)	
3’	2.41 (ddd, J = 12.3, 10.2, 3.9 Hz)	65.5	3.3 (m)	64.7
4’ (proS)	1.07 (m)	30.5	1.44 (m)	30.48
4’ (proR)	1.58 (m)		1.91 (m)	
5’	3.19 (m)	69.6	3.68 (m)	67.83
6’	1.07 (m)	22	1.03 (m)	19.5
7’,8’	2.21 (s)	40.7	2.68 (s)<br>2.73 (s)	40.7<br>37.5
1”	4.77 (d, J = 5 Hz)	95.8		
2” (proS)	1.53 (m)	34.8		
2” (proR)	2.28 (d, J = 15 Hz)			
3”		72.5		
4”	2.92 (dd, J = 9.4, 7.3 Hz)	77.5		
5”	4.03 (m)	66.8		
6”	1.13 (brs)	20.8		
7”	1.09 (s)	21.5		
8”	3.21 (s)	48.8		

Supplementary Table 10: Effect of *llmA* expression on susceptibility to antibiotics that target the peptidyltransferase center.

**Antibiotic**	***E. coli*** **BL21(DE3) pET11a MIC** (μg/mL)	***E. coli*** **BL21(DE3) pET11a-*llmA* MIC** (μg/mL)
Linezolid	16	2
Flopristin	16	16
Clindamycin	32-64	1024
Chloramphenicol	4	2
Tiamulin	128	32
Erythromycin	32	32
Telithromycin	4	4

Supplementary Table 11: List of *Paenibacillus* strains and assembly IDs used for generating the *Paenibacillus* species tree.

Strain	Assembly Accession	Strain	Assembly Accession
Paenibacillus alvei DSM 29	GCF 000293805.1	*Paenibacillus sabinae* T27	GCF 000612505.1
Paenibacillus assamensis DSM	GCF 000422445.1	*Paenibacillus senegalensis* JC66	GCF 000285515.1
Paenibacillus borealis	GCF 000758665.1	*Paenibacillus sonchi* X19-5	GCF 000316285.1
Paenibacillus chitinolyticus NBRC	GCF 000739915.1	*Paenibacillus* sp. 1-18	GCF 000520755.1
Paenibacillus curdlanolyticus YK9	GCF 000179615.1	*Paenibacillus* sp. 1-49	GCF 000520815.1
Paenibacillus daejeonensis DSM	GCF 000378385.1	*Paenibacillus* sp. A9	GCF 000346635.1
Paenibacillus dauci	GCF 000971985.1	*Paenibacillus* sp. FSL H7-0357	GCF 000758525.1
Paenibacillus durus ATCC 35681	GCF 000993825.1	*Paenibacillus* sp. FSL H7-0737	GCF 000758545.1
Paenibacillus ehimensis A2	GCF 000612225.1	*Paenibacillus* sp. FSL H7-689	GCF 000517905.1
Paenibacillus elgii B69	GCF 000213315.1	*Paenibacillus* sp. FSL H8-237	GCF 000517925.1
Paenibacillus fonticola DSM	GCF 000381905.1	*Paenibacillus* sp. FSL H8-457	GCF 000517945.1
Paenibacillus forsythiae T98	GCF 000520735.1	*Paenibacillus* sp. FSL P4-0081	GCF 000758565.1
Paenibacillus ginsengihumi DSM	GCF 000380965.1	*Paenibacillus* sp. FSL R5-0345	GCF 000758585.1
Paenibacillus graminis	GCF 000758705.1	*Paenibacillus* sp. FSL R5-0912	GCF 000758605.1
Paenibacillus harenae DSM	GCF 000422465.1	*Paenibacillus* sp. FSL R5-192	GCF 000517845.1
Paenibacillus lactis 154	GCF 000230915.1	*Paenibacillus* sp. FSL R5-808	GCF 000517885.1
Paenibacillus macerans	GCF 000746875.1	*Paenibacillus* sp. FSL R7-0273	GCF 000758625.1
Paenibacillus massiliensis T7	GCF 000520695.1	*Paenibacillus* sp. FSL R7-0331	GCF 000758645.1
Paenibacillus mucilaginosus K02	GCF 000258535.2	*Paenibacillus* sp. FSL R7-269	GCF 000517865.1
Paenibacillus mucilaginosus	GCF 000218915.1	*Paenibacillus* sp. FSL R7-277	GCF 000517965.1
Paenibacillus odorifer	GCF 000758725.1	*Paenibacillus* sp. G4	GCF 000723885.1
Paenibacillus panacisoli DSM	GCF 000426545.1	*Paenibacillus* sp. HGF5	GCF 000204455.1
Paenibacillus pasadenensis DSM	GCF 000422485.1	*Paenibacillus* sp. HGH0039	GCF 000411255.1
Paenibacillus peoriae KCTC 3763	GCF 000236805.1	*Paenibacillus* sp. HW567	GCF 000374185.1
Paenibacillus pinihumi DSM	GCF 000422505.1	*Paenibacillus* sp. IHB B 3415	GCF 000802655.2
Paenibacillus polymyxa	GCF 000785455.1	*Paenibacillus* sp. JDR-2	GCF 000023585.1
Paenibacillus polymyxa 1-43	GCF 000520795.1	*Paenibacillus* sp. MAEPY1	GCF 000499305.1
Paenibacillus polymyxa A18	GCF 000809185.1	*Paenibacillus* sp. MAEPY2	GCF 000499205.1
Paenibacillus polymyxa ATCC 842	GCF 000217775.1	*Paenibacillus* sp. MSt1	GCF 000722545.1
Paenibacillus polymyxa CR1	GCF 000507205.3	*Paenibacillus* sp. oral taxon 786	GCF 000159955.1
Paenibacillus polymyxa E681	GCF 000146875.1	*Paenibacillus* sp. OSY-SE	GCF 000283315.1
Paenibacillus polymyxa M1	GCF 000237325.1	*Paenibacillus* sp. PAMC 26794	GCF 000316035.1
Paenibacillus polymyxa OSY-DF	GCF 000265445.1	*Paenibacillus* sp. UNC451MF	GCF 000686845.1
Paenibacillus polymyxa SC2	GCF 000164985.1	*Paenibacillus* sp. UNCCL52	GCF 000686825.1
Paenibacillus polymyxa SQR-21	GCF 000597985.1	*Paenibacillus* sp. URHA0014	GCF 000620565.1
Paenibacillus riograndensis SBR5	GCF 000981585.1	*Paenibacillus* sp. Y412MC10	GCF 000024685.1
Paenibacillus terrigena DSM	GCF 000374845.1	*Paenibacillus stellifer*	GCF 000758685.1
Paenibacillus vortex V453	GCF 000193415.1	*Paenibacillus taiwanensis* DSM	GCF 000425125.1
Paenibacillus wynnii	GCF 000757885.1	*Paenibacillus terrae*	GCF 000943545.1
Paenibacillus terrae HPL-003	GCF 000235585.1		

Supplementary Table 12: Oligonucleotides used in this study.

Gene	Vector	Primer Direction	Restriction Site	Primer (5' to 3')
mphI	pET22a	Forward	NdeI	ATTAGTcatatgACAATAGCAAAACCAAATGATGATTATACACAAGTG
		Reverse	XhoI	TCCTTctcgagCTCCGTCAAACCCAGAGCATTCC
vatI	pET28a	Forward	NheI	TACTGAgctagcATGACAGGACCGAATCC
		Reverse	BamHI	TCAGTAggatccCTAATTTTGTCTCATTTTTCTAAGC
vgbC	pET28a	Forward	NheI	AGACTgctagcATGCAGATCGCCGCACAGGAGTACAAGACAGC
		Reverse	XhoI	AATActcgagTTAATACTGAATCGTAAACCGGCCG
rphB	pET28a	Forward	NheI	TACTGAgctagcATGCGTTCGTTGGTTCTCG
		Reverse	EcoRI	TCAGTAgaattcCTATGACAATATTTCAATATAGCCTTCGGACC
cpaA	pET28a	Forward	NheI	TACTGAgctagcCTTCGTATCACGGCCATGACC
		Reverse	BamHI	TCAGTAggatccTTACAATCCGGAGCCCGG
llmA	pET11a	Forward	NheI	TACTGAgctagcATGTATGTAGCTCAGGATTGGC
		Reverse	EcoRI	TCAGTAgaattcTCAGGACTCCCATCGACC
ileRS	pET11a	Forward	NheI	TACTGAgctagcATGAACAGAGTAGACGTCAAGGAGAAGGC
		Reverse	BamHI	TCAGTAggatccTTAGGCGATGTGGATGCCG
bahA	pET11a	Forward	NheI	TACTGAgctagcGAAATACACAAAGAGAAAGAAAAAGAAAAAGGCG
		Reverse	BamHI	TCAGTAggatccTTAAGATCGAATGCCAACATCACG
bahA (C-terminal hydrolase domain)	pET11a	Forward	NheI	TACTGAgctagcAACAATCCGGCCGAACAAGG
		Reverse	BamHI	TCAGTAggatccTTAAGATCGAATGCCAACATCACG
	pET21a (sub-cloned from pET11a)	Forward	XbaI	ATTCCCCtctagaAATAATTTTGTTTAACTTTAAGAAGG
		Reverse	XhoI	TCAGTActcgagAGATCGAATGCCAACATCACG
bahA (Transmembrane domain)	pET11a	Forward	NheI	TACTGAgctagcGAAATACACAAAGAGAAAGAAAAAGAAAAAGGCG
		Reverse	BamHI	TCAGTAggatccCGTCGAGTCTTCATATCGGGTTG
tetAB(48)	pUC19	Forward	KpnI	TACTAggtaccTCCATGGAGATGTGTTCCGAGG
		Reverse	XbaI	TAGTAtctagaGACAGGATGGGTAACGCCG
aac(2')-IIb	pET21a/pET28a	Forward	NheI	TACTGAgctagcATGAACCATAGGAAAGGTAACG
		Reverse	BamHI	TCAGTAggatccTCAACTGATCGTGAAGTTAACTCC
aac(6’)-34	pET21a/pET28a	Forward	NheI	TACTGAgctagcATGAGAATTGGGGATCTAATAAGGG
		Reverse	BamHI	TCAGTAggatccTTACTTTCGCTCTGCCCG
taeA	pUC19	Forward	KpnI	TACTAggtaccCGGAGTTAGGCAGCCTTAGCC
		Reverse	XbaI	TAGTAtctagaACTAACAAAGGGGCGTCCCG
catU	pET28a	Forward	NdeI	TGCATGCAcatatgAAGTTTCACATTATTAATGTTGAAGAGTGG
		Reverse	XhoI	TGTAATCctcgagAGTTAACCAATCTTCAAAAGAATCCGCC
